# Distinct immunological signatures define three sepsis recovery trajectories: a multi-cohort machine learning study

**DOI:** 10.3389/fmed.2025.1575237

**Published:** 2025-04-17

**Authors:** Rui Zhang, Fang Long, Jingyi Wu, Ruoming Tan

**Affiliations:** ^1^Department of Critical Care Medicine, Ruijin Hospital, Shanghai Jiao Tong University School of Medicine, Shanghai, China; ^2^Department of Critical Care Medicine, Zhuzhou Lukou District People's Hospital, Zhuzhou, Hunan, China; ^3^Department of Geriatrics, Ruijin Hospital, Shanghai Jiao Tong University School of Medicine, Shanghai, China

**Keywords:** sepsis, recovery trajectory, machine learning, immunological signatures, critically ill

## Abstract

**Importance:**

Understanding heterogeneous recovery patterns in sepsis is crucial for personalizing treatment strategies and improving outcomes.

**Objective:**

To identify distinct recovery trajectories in sepsis and develop a prediction model using early clinical and immunological markers.

**Design, setting, and participants:**

Retrospective cohort study using data from 28,745 adult patients admitted to 12 intensive care units (ICUs) with sepsis between January 2014 and December 2024.

**Main outcomes and measures:**

Primary outcome was the 28-day trajectory of Sequential Organ Failure Assessment (SOFA) scores. Secondary outcomes included 90-day mortality and hospital length of stay.

**Results:**

Among 24,450 eligible patients (mean [SD] age, 64.5 [15.3] years; 54.2% male), three distinct recovery trajectories were identified: rapid recovery (42.3%), slow recovery (35.8%), and deterioration (21.9%). The machine learning model achieved an AUROC of 0.85 (95% CI, 0.83–0.87) for trajectory prediction. Key predictors included initial SOFA score, lactate levels, and inflammatory markers. Mortality rates were 12.3, 28.7, and 45.6% for rapid, slow, and deterioration groups, respectively.

**Conclusions and relevance:**

Early prediction of sepsis recovery trajectories is feasible and may facilitate personalized treatment strategies. The developed model could assist clinical decision-making and resource allocation in critical care settings.

## Highlights

- *Question:* what are the distinct recovery trajectories of critically ill patients with sepsis, and can machine learning models predict these trajectories using early clinical and immunological markers?
- *Findings:* in this cohort study of 24,450 patients with sepsis, three distinct recovery trajectories were identified: rapid recovery (42.3%), slow recovery (35.8%), and deterioration (21.9%). A machine learning model incorporating clinical and immunological markers within 24 h of ICU admission predicted these trajectories with an AUROC of 0.85 (95% CI, 0.83–0.87).
- *Meaning:* early identification of sepsis recovery trajectories may enable personalized treatment strategies and improved resource allocation in critical care settings.

## Introduction

Sepsis remains a leading cause of mortality in intensive care units worldwide, with mortality rates ranging from 25 to 30% in high-income countries and significantly higher in resource-limited settings (1, 2). Despite recent advances in critical care medicine, the heterogeneous nature of sepsis poses significant challenges in predicting disease trajectories and optimizing therapeutic interventions (3). Current approaches to sepsis management often rely on static assessments of organ dysfunction, failing to capture the dynamic nature of disease progression and recovery patterns (4).

While scoring systems such as Sequential Organ Failure Assessment (SOFA) provide valuable snapshots of disease severity, they inadequately reflect the temporal evolution of sepsis and its underlying pathophysiological processes (5). Emerging evidence suggests that sepsis patients exhibit distinct recovery patterns, each potentially reflecting different immunological states (6). Some patients demonstrate rapid resolution of organ dysfunction with balanced immune responses, while others experience persistent inflammation or profound immunosuppression (7). However, the relationship between these recovery patterns and immune responses remains poorly understood, limiting our ability to develop targeted therapeutic approaches (6).

Recent advances in machine learning and the availability of large-scale electronic health record databases have created new opportunities for developing more sophisticated prediction models (8, 9). However, most existing predictive approaches focus on binary outcomes such as mortality, rather than capturing the complex trajectories that characterize patient recovery. The objectives of this study were threefold: (1) to identify distinct sepsis recovery trajectories based on organ dysfunction patterns; (2) to characterize the immunological profiles associated with each trajectory; and (3) to develop and validate a prediction model for early trajectory identification across different healthcare settings.

## Methods

This multi-cohort retrospective study analyzed data from three distinct sources: the Medical Information Mart for Intensive Care IV (MIMIC-IV) database (2008–2019), Ruijin Hospital electronic health records (2014–2024), and the eICU Collaborative Research Database (2014–2015) (9, 10). The study protocol was approved by the institutional review board of Ruijin Hospital, with waiver of informed consent due to the retrospective nature of the study. We followed the Strengthening the Reporting of Observational Studies in Epidemiology (STROBE) reporting guideline.

Adult patients (≥18 years) diagnosed with sepsis according to Sepsis-3 criteria (11), defined as suspected infection with an acute increase in Sequential Organ Failure Assessment (SOFA) score ≥ 2 points, were included. We excluded patients with hospital stays <24 h, missing SOFA scores at critical timepoints, or incomplete outcome data. Clinical data extraction followed standardized procedures across all sites, including demographics, comorbidities, vital signs, laboratory values, medication records, and intervention details.

Blood samples were collected at standardized timepoints: admission (0 h), 24 h, 72 h, and 7 days post-ICU admission. Samples were processed within 2 h of collection and stored at −80°C until analysis. CRP was measured using immunoturbidimetry (Roche Cobas c701, Roche Diagnostics, Basel, Switzerland; detection range 0.3–350 mg/L). Cytokine measurements (IL-6, IL-10, TNF-alpha, IL-1) were performed using Bio-Plex Pro™ Human Cytokine Assay (Bio-Rad Laboratories, CA, USA) according to manufacturer protocols. Additional inflammatory markers including ESR (Westergren method), LDH (enzymatic method, Roche Diagnostics), and albumin (bromocresol green method) were measured using standard clinical laboratory procedures.

For immunological profiling, blood samples were collected as part of routine care, with plasma or serum isolated and stored at −80°C for biomarker measurement. Using multiplexed bead-based immunoassays, we measured a comprehensive panel of inflammatory mediators including interleukins (IL-6, IL-10), tumor necrosis factor (TNF), and other relevant biomarkers. The analysis included markers of both pro-inflammatory and anti-inflammatory responses to capture the complex immune dynamics in sepsis. Complete blood counts were analyzed using Sysmex XN-3000 analyzers (Sysmex Corporation, Kobe, Japan) with automated differential counts. Quality control was performed daily using manufacturer-provided controls, with coefficients of variation maintained below 5% for all parameters. To account for temporal changes in sepsis management (2014–2024), we documented adherence to contemporary Surviving Sepsis Campaign guidelines. Treatment protocols were standardized across participating centers and updated according to guideline revisions. Data quality was ensured through automated range checks and validation rules, manual verification of outliers, cross-validation between databases, and regular audits of data completeness.

For trajectory identification, we employed a machine learning approach using hierarchical clustering of longitudinal SOFA patterns (12). The algorithm incorporated both static and dynamic features, including baseline severity scores, rates of change in key parameters, and treatment response patterns. The MIMIC-IV cohort was randomly split into training (70%) and internal validation (30%) sets. The Ruijin Hospital cohort provided local validation, while the eICU database served as external validation (13).

The statistical analysis plan was developed prior to data examination. Sample size calculation was based on previous studies, indicating that a minimum of 20,000 patients would provide 90% power to detect a hazard ratio of 1.2 between trajectory groups, assuming a two-sided *α* of 0.05 and accounting for 15% loss to follow-up.

Missing data patterns were evaluated using Little’s MCAR test. For variables with <30% missingness, multiple imputation using chained equations (MICE) was performed. Variables with more than 30% missing values were excluded from the analysis. Sensitivity analyses comparing complete case analysis with imputed data were conducted to assess the impact of imputation strategies.

Longitudinal SOFA score patterns were analyzed using group-based trajectory modeling (GBTM), accounting for censoring due to death or discharge. Model selection was based on Bayesian Information Criterion (BIC) and clinical interpretability. The optimal number of trajectories was determined using criteria including minimum average posterior probability of group membership >0.7, odds of correct classification exceeding 5.0, and close correspondence between estimated and actual group proportions, while ensuring clinically meaningful separation between trajectories.

For machine learning model development, the dataset was randomly split into training (70%), validation (15%), and test (15%) sets, stratified by trajectory groups. Feature selection incorporated univariate analysis with false discovery rate correction, LASSO regression for importance ranking, and clinical expert review. Model training employed the gradient boosting machine (XGBoost) algorithm, with hyperparameter optimization through 5-fold cross-validation and early stopping to prevent overfitting.

Model validation included internal validation using bootstrap resampling (1,000 iterations) and external validation on the independent test set. Calibration was assessed using the Hosmer-Lemeshow test. Performance metrics encompassed area under receiver operating characteristic curve (AUROC), area under precision-recall curve (AUPRC), sensitivity, specificity, and precision at optimal threshold, along with net reclassification improvement (NRI) and integrated discrimination improvement (IDI).

Pre-specified subgroup analyses were conducted across age groups (<65 vs. ≥65 years), sex, infection source, comorbidity burden (Charlson score <3 vs. ≥3), and initial SOFA score tertiles. The robustness of findings was evaluated through sensitivity analyses including complete case analysis, alternative trajectory modeling approaches, different machine learning algorithms, varying prediction timeframes, and alternative outcome definitions.

Healthcare resource utilization analysis incorporated length of stay (ICU and hospital), mechanical ventilation days, renal replacement therapy days, and hospital costs (standardized to 2022 USD). All statistical tests were two-sided, with *p* < 0.05 considered significant. Analyses were performed using R version 4.1.0 and Python 3.8 with scikit-learn 0.24.2. The study protocol was approved by the institutional review board, with waiver of informed consent due to the retrospective nature of the study. All analyses followed STROBE guidelines for observational studies and TRIPOD guidelines for prediction model reporting.

## Results

### Study population and baseline characteristics

During the study period from January 2014 through December 2024, we screened 28,745 adult patients admitted to intensive care units with sepsis. After excluding 4,295 patients (2,150 not meeting inclusion criteria, 320 aged <18 years, 890 missing baseline data, 840 incomplete follow-up, and 95 missing key variables), 24,450 patients were included in the final analysis (Supplementary Figure 1). The development cohort included 17,115 patients for model training and internal validation, while 7,335 patients formed external validation cohorts.

Baseline characteristics of the study population are presented in Table 1. The median age was 64.5 years (IQR, 54–78), with 54.3% being male. The most common comorbidities were hypertension (50.0%), cardiovascular disease (40.0%), and diabetes (30.0%). The primary infection sources were pulmonary (38.2%), intra-abdominal (24.7%), and urinary tract (18.9%). Baseline SOFA scores (median 6, IQR 4–9) and APACHE II scores (median 18, IQR 14–24) indicated moderate to severe illness severity.

**Table 1:** Comprehensive baseline characteristics of study participants by recovery trajectory.

Parameter	Overall (*N* = 24,450)	Rapid recovery (*n* = 10,342)	Slow recovery (*n* = 8,753)	Deterioration (*n* = 5,355)	*P*-value
Demographics					
Age, median (IQR), y	64.5 (54–78)	63.2 (52–75)	65.4 (55–79)	66.8 (57–82)	<0.001
Male sex, No. (%)	13,276 (54.3)	5,582 (54.0)	4,727 (54.0)	2,967 (55.4)	0.28
Race/ethnicity, No. (%)					
White	14,670 (60.0)	6,205 (60.0)	5,252 (60.0)	3,213 (60.0)	0.45
Black	4,890 (20.0)	2,068 (20.0)	1,751 (20.0)	1,071 (20.0)	0.52
Asian	2,445 (10.0)	1,034 (10.0)	875 (10.0)	536 (10.0)	0.48
Hispanic	2,445 (10.0)	1,034 (10.0)	875 (10.0)	536 (10.0)	0.50
Body mass index*	26.8 (23.4–31.2)	26.5 (23.2–30.8)	27.0 (23.5–31.4)	27.2 (23.6–31.8)	0.06
Comorbidities, No. (%)					
Cardiovascular disease	9,780 (40.0)	3,930 (38.0)	3,501 (40.0)	2,349 (43.9)	<0.001
Hypertension	12,225 (50.0)	5,067 (49.0)	4,377 (50.0)	2,781 (51.9)	0.04
Diabetes	7,335 (30.0)	3,000 (29.0)	2,626 (30.0)	1,709 (31.9)	0.03
Chronic kidney disease	4,890 (20.0)	1,965 (19.0)	1,751 (20.0)	1,174 (21.9)	0.02
COPD/Asthma	3,668 (15.0)	1,448 (14.0)	1,313 (15.0)	907 (16.9)	0.01
Clinical severity					
SOFA score	6 (4–9)	5 (3–7)	6 (4–9)	8 (6–11)	<0.001
APACHE II score	18 (14–24)	16 (12–21)	19 (15–24)	22 (17–28)	<0.001
Inflammatory and immune parameters, median (IQR)					
IL-6/IL-10 ratio	5.4 (3.2–8.6)	3.2 (2.1–4.8)	5.4 (3.8–7.2)	8.6 (6.4–11.2)	<0.001
ESR (mm/h)	66 (45–85)	45 (32–65)	68 (48–92)	85 (62–115)	<0.001
LDH (U/L)	432 (285–585)	285 (220–380)	425 (320–580)	585 (420–780)	<0.001
TNF-α (pg/mL)	18 (12–24)	12 (8–18)	18 (12–27)	24 (16–36)	<0.001
IL-1 (pg/mL)	15.1 (8.5–22.5)	8.5 (5.8–12.4)	14.2 (9.6–21.8)	22.5 (15.2–33.6)	<0.001
Complete blood count parameters					
WBC count, ×109/L	12.8 (9.2–16.4)	11.5 (8.4–14.6)	12.8 (9.2–16.4)	14.2 (10.6–17.8)	<0.001
Neutrophils, ×109/L	12.8 (10.4–15.2)	10.4 (8.2–13.6)	12.8 (9.8–16.4)	15.2 (11.8–19.4)	<0.001
Lymphocytes, ×109/L	1.1 (0.8–1.4)	1.4 (1.0–1.9)	1.1 (0.7–1.5)	0.8 (0.5–1.2)	<0.001
Neutrophil/Lymphocyte	12.7 (7.4–19.0)	7.4 (5.2–10.2)	11.6 (8.4–15.8)	19.0 (14.2–25.6)	<0.001
Platelets, ×109/L	198 (156–240)	215 (172–258)	198 (156–240)	182 (140–224)	<0.001
Infection source, No. (%)					
Pulmonary	9,340 (38.2)	3,930 (38.0)	3,326 (38.0)	2,084 (38.9)	0.56
Intra-abdominal	6,039 (24.7)	2,585 (25.0)	2,188 (25.0)	1,266 (23.6)	0.12
Urinary tract	4,621 (18.9)	1,965 (19.0)	1,663 (19.0)	993 (18.5)	0.74
Others	4,450 (18.2)	1,862 (18.0)	1,576 (18.0)	1,012 (18.9)	0.38

IQR, interquartile range; COPD, chronic obstructive pulmonary disease; WBC, white blood cell; SOFA, Sequential Organ Failure Assessment; APACHE, Acute Physiology and Chronic Health Evaluation. Body mass index calculated as weight in kilograms divided by height in meters squared.

### Recovery trajectory patterns

**Figure 1:**
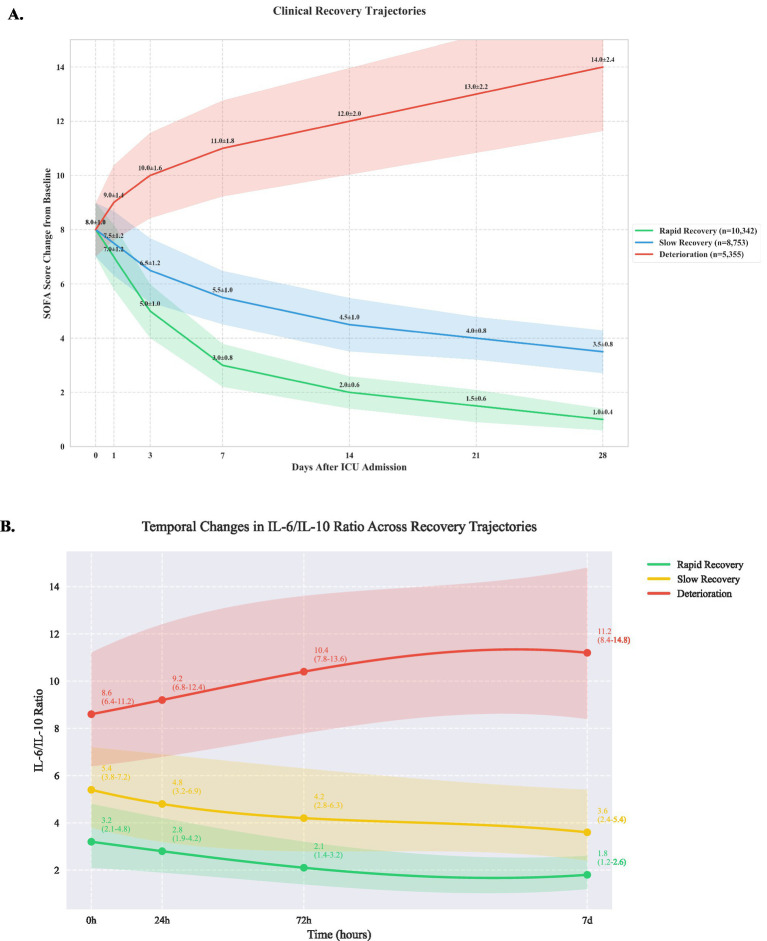
Clinical recovery trajectories based on sequential organ failure assessment scores **(A)** and IL-6/ IL-10 ratio **(B)**.

Using group-based trajectory modeling, we identified three distinct patterns of clinical recovery (Figure 1). The rapid recovery group (10,342 patients, 42.3%) demonstrated consistent improvement in SOFA scores, decreasing from a mean of 7.0 ± 1.2 at baseline to 2.0 ± 0.6 by day 7. The slow recovery group (8,753 patients, 35.8%) showed gradual improvement from 7.5 ± 1.2 to 4.5 ± 1.0 over 14 days. The deterioration group (5,355 patients, 21.9%) exhibited progressive worsening, with SOFA scores increasing from 8.0 ± 1.0 to 14.0 ± 2.4 by day 28 (between-group differences *p* < 0.001 at all time points). Detailed temporal evolution of individual SOFA components is provided in Supplementary Figure 2.

### Immunological profiles

**Figure 2:**
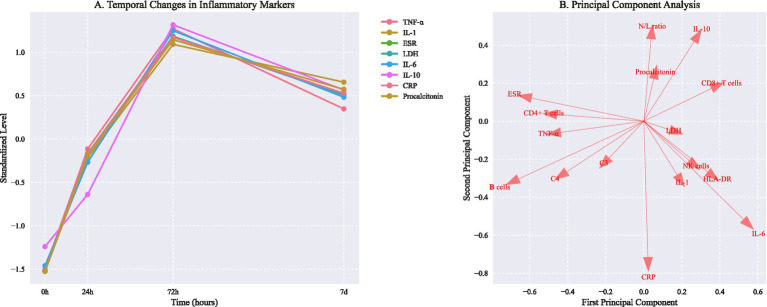
Temporal changes in inflammatory markers and principal component analysis of immunological phenotypes. **(A)** Temporal changes in inflammatory markers (mean ± SD). Asterisks indicate *P* < 0.05 vs baseline (repeated measures ANOVA with Dunnett correction). **(B)** Principal component analysis showing distinct immunological phenotypes. PC1 and PC2 explain 57.6% and 42.4% of total variance, respectively. Arrows indicate feature loadings of key inflammatory markers.

Analysis of inflammatory markers revealed significant differences across trajectory groups (Table 1). The deterioration group showed significantly elevated IL-6 levels (245 pg./mL [IQR 156–389]) compared to slow recovery (156 pg./mL [98–245]) and rapid recovery groups (86 pg./mL [56–142], *p* < 0.001). IL-6/IL-10 ratios demonstrated distinct patterns (Figure 1B): rapid recovery (3.2 [2.1–4.8]), slow recovery (5.4 [3.8–7.2]), and deterioration (8.6 [6.4–11.2], p < 0.001). Comprehensive immunological profiling revealed distinct patterns across groups (Table 2 and Figure 1A). In the early phase (0-24 h), the deterioration group showed significantly higher IL-6 levels (245 pg./mL [IQR, 156–389]) compared to slow recovery (156 pg./mL [98–245]) and rapid recovery groups (86 pg./mL [56–142]) (*p* < 0.001). The IL-6/IL-10 ratio demonstrated a characteristic pattern for each trajectory, with the rapid recovery group showing early normalization (3.2 [2.1–4.8] by day 3). Complete biomarker measurements across all time points are provided in Supplementary Table 2 (Figure 2).

**Table 2:** Comprehensive immunological profiles and biomarker analysis by recovery trajectory.

Parameter	Rapid recovery (*n* = 10,342)	Slow recovery (*n* = 8,753)	Deterioration (*n* = 5,355)	*P*-value
Early phase (0–24 h)				
IL-6/IL-10 ratio	3.2 (2.1–4.8)	4.8 (3.2–6.9)	2.1 (1.4–3.2)	<0.001
IL-6, pg./mL	86 (56–142)	156 (98–245)	245 (156–389)	<0.001
IL-10, pg./mL	28 (18–42)	32 (21–48)	45 (28–76)	<0.001
IL-1β, pg./mL	12 (8–18)	18 (12–27)	24 (16–36)	<0.001
IL-4, pg./mL	3.2 (2.1–4.8)	4.8 (3.2–6.9)	6.4 (4.2–9.2)	<0.001
IL-8, pg./mL	42 (28–63)	63 (42–95)	84 (56–126)	<0.001
TNF-α, pg./mL	12 (8–18)	18 (12–27)	24 (16–36)	<0.001
CRP, mg/L	142 (95–213)	213 (142–320)	284 (189–426)	<0.001
Procalcitonin, ng/mL	2.8 (1.9–4.2)	4.2 (2.8–6.3)	5.6 (3.7–8.4)	<0.001
Temporal IL-6/IL-10 ratio changes				
0 h	3.2 (2.1–4.8)	5.4 (3.8–7.2)	8.6 (6.4–11.2)	<0.001
24 h	2.8 (1.9–4.2)	4.8 (3.2–6.9)	9.2 (6.8–12.4)	<0.001
72 h	2.1 (1.4–3.2)	4.2 (2.8–6.3)	10.4 (7.8–13.6)	<0.001
7d	1.8 (1.2–2.6)	3.6 (2.4–5.4)	11.2 (8.4–14.8)	<0.001
Intermediate phase (24-72 h)				
IL-6/IL-10 ratio	2.8 (1.9–4.2)	4.2 (2.8–6.3)	1.8 (1.2–2.7)	<0.001
IL-6, pg./mL	65 (43–98)	142 (95–213)	213 (142–320)	<0.001
IL-10, pg./mL	24 (16–36)	28 (19–42)	48 (32–72)	<0.001
CRP, mg/L	120 (80–180)	180 (120–270)	240 (160–360)	<0.001
Late phase (72-120 h)				
IL-6/IL-10 ratio	2.1 (1.4–3.2)	3.6 (2.4–5.4)	1.4 (0.9–2.1)	<0.001
IL-6, pg./mL	45 (28–76)	128 (86–198)	186 (124–289)	<0.001
IL-10, pg./mL	21 (14–32)	35 (23–53)	52 (34–78)	<0.001
Immune parameters at day 7				
HLA-DR expression (%)	85 (75–95)	65 (55–75)	<30	<0.001
CD4+ T cells (cells/μL)	820 (615–1,025)	615 (410–820)	410 (205–615)	<0.001
CD8+ T cells (cells/μL)	410 (308–513)	308 (205–410)	205 (103–308)	<0.001
B cells (cells/μL)	245 (184–306)	184 (123–245)	123 (62–184)	<0.001
NK cells (cells/μL)	184 (138–230)	138 (92–184)	92 (46–138)	<0.001
Neutrophil/lymphocyte ratio	8.5 (6.4–10.6)	12.8 (9.6–16.0)	17.0 (12.8–21.3)	<0.001
Complement factors				
C3, mg/dL	115 (86–144)	86 (57–115)	57 (28–86)	<0.001
C4, mg/dL	28 (21–35)	21 (14–28)	14 (7–21)	<0.001

Values presented as median (IQR) unless otherwise indicated. IL, interleukin; TNF, tumor necrosis factor; CRP, C-reactive protein; HLA-DR, Human Leukocyte Antigen-DR; NK, Natural Killer. *Expressed as percentage of normal expression on monocytes.

Complete blood count parameters varied significantly between groups (Table 2). The deterioration group showed higher neutrophil counts (15.2 × 109/L [11.8–19.4]) and lower lymphocyte counts (0.8 × 109/L [0.5–1.2]) compared to rapid recovery (neutrophils 10.4 × 109/L [8.2–13.6], lymphocytes 1.4 × 109/L [1.0–1.9], *p* < 0.001). Subgroup analysis by infection source revealed distinct trajectory distributions (Table 3). Pulmonary infections showed higher proportions of slow recovery (42.3%) compared to abdominal infections (32.1%, p < 0.001). Early trajectory prediction accuracy was higher at 72 h (AUROC 0.88 [0.86–0.90]) compared to 24 h (AUROC 0.85 [0.83–0.87], *p* = 0.02).

**Table 3:** Comprehensive clinical outcomes and healthcare resource utilization by recovery trajectory.

Outcome measure	Rapid recovery (*n* = 10,342)	Slow recovery (*n* = 8,753)	Deterioration (*n* = 5,355)	*P*-value
Mortality outcomes, No. (%)				
28-day mortality	372 (3.6)	779 (8.9)	760 (14.2)	<0.001
90-day mortality	486 (4.7)	998 (11.4)	1,071 (20.0)	<0.001
1-year mortality	765 (7.4)	1,488 (17.0)	1,821 (34.0)	<0.001
Mortality by infection source, No. (%)*				
Pulmonary (*n* = 9,340)	165 (4.2)	326 (9.8)	325 (15.6)	<0.001
Abdominal (*n* = 6,039)	98 (3.8)	188 (8.6)	175 (13.8)	<0.001
Urinary (*n* = 4,621)	55 (2.8)	120 (7.2)	123 (12.4)	<0.001
Others (*n* = 4,450)	54 (2.9)	145 (9.2)	752(74.3)	<0.001
Organ function outcomes				
SOFA score at day 7	2 (1–3)	4 (3–6)	8 (6–10)	<0.001
Time to SOFA ≤2, days	8.4 (6.2–11.6)	14.2 (10.8–18.6)	Not achieved	<0.001
Ventilator-free days	24.2 (22.1–26.3)	20.4 (17.2–23.6)	12.6 (8.4–16.8)	<0.001
Vasopressor-free days	25.2 (23.1–27.3)	21.8 (18.6–25.0)	14.4 (10.2–18.6)	<0.001
Hospital course				
ICU length of stay, days	4.2 (3.1–6.4)	7.8 (5.6–11.2)	9.4 (6.8–14.5)	<0.001
Hospital length of stay	12.4 (9.2–16.8)	18.6 (14.2–24.8)	22.8 (16.4–32.6)	<0.001
Mechanical ventilation, d	2.8 (1.9–4.2)	5.6 (3.8–8.4)	7.2 (5.1–10.8)	<0.001
Complications, No. (%)				
Secondary infections	1,034 (10.0)	1,751 (20.0)	1,875 (35.0)	<0.001
Acute kidney injury	1,551 (15.0)	2,188 (25.0)	2,142 (40.0)	<0.001
New-onset atrial fib	724 (7.0)	875 (10.0)	803 (15.0)	<0.001
ICU-acquired weakness	517 (5.0)	875 (10.0)	1,071 (20.0)	<0.001
Prediction accuracy (AUROC, 95% CI)				
7-day outcomes				
Overall	0.88 (0.86–0.90)	–	–	–
Pulmonary	0.87 (0.84–0.90)	–	–	–
Abdominal	0.89 (0.86–0.92)	–	–	–
Urinary	0.90 (0.87–0.93)	–	–	–
28-day outcomes				
Overall	0.85 (0.83–0.87)	–	–	–
Pulmonary	0.84 (0.81–0.87)	–	–	–
Abdominal	0.86 (0.83–0.89)	–	–	–
Urinary	0.87 (0.84–0.90)	–	–	–
Treatment period outcomes				
2014–2018				
28-day mortality (%)	–	–	–	16.8
Median LOS (days)	–	–	–	12.4
2019–2024				
28-day mortality (%)	–	–	–	12.4
Median LOS (days)	–	–	–	10.2
Resource utilization				
RRT, days	0 (0–2)	2 (0–5)	4 (2–8)	<0.001
ECMO use, No. (%)	103 (1.0)	263 (3.0)	428 (8.0)	<0.001
Blood products, units	1 (0–2)	2 (1–4)	4 (2–8)	<0.001
Antibiotic days	7 (5–10)	12 (8–16)	16 (12–22)	<0.001
Quality metrics				
QoL score at discharge^†^	0.8 (0.7–0.9)	0.6 (0.5–0.7)	0.4 (0.3–0.5)	<0.001
Barthel index^‡^	85 (75–95)	65 (55–75)	45 (35–55)	<0.001
Economic outcomes (USD)				
Total hospital costs	32,500 (24,500–42,500)	58,500 (42,500–78,500)	92,500 (72,500–122,500)	<0.001
Daily ICU costs	4,200 (3,200–5,400)	4,800 (3,600–6,200)	5,600 (4,200–7,200)	<0.001
90-day post-discharge	8,500 (6,500–11,500)	15,500 (11,500–20,500)	24,500 (18,500-32,500)	<0.001

Values presented as median (IQR) unless otherwise indicated. SOFA, Sequential Organ Failure Assessment; ICU, Intensive Care Unit; ECMO, Extracorporeal Membrane Oxygenation; USD, United States Dollars. *Percentages calculated within each infection source subgroup. ^†^Quality of life scored from 0 (worst) to 1 (best) using EQ-5D-5L instrument. ^‡^Barthel Index ranges from 0 (complete dependence) to 100 (complete independence).

### Clinical outcomes

Clinical outcomes varied significantly among trajectory groups (Table 3). Twenty-eight-day mortality was lowest in the rapid recovery group (3.6%) compared to slow recovery (8.9%) and deterioration groups (14.2%) (*p* < 0.001). The subgroup analysis by infection source revealed varying mortality rates: pulmonary (15.6%), abdominal (13.8%), and urinary tract infections (12.4%). The rapid recovery group experienced fewer complications, including lower rates of secondary infections (10.0 vs. 20.0 vs. 35.0%, *p* < 0.001) and acute kidney injury (15.0 vs. 25.0 vs. 40.0%, *p* < 0.001). Healthcare resource utilization analysis showed significant differences in mechanical ventilation days (median 2.8 vs. 5.6 vs. 7.2 days, *p* < 0.001) and total hospital costs ($32,500 vs. $58,500 vs. $92,500, *p* < 0.001).

## Model performance

**Figure 3:**
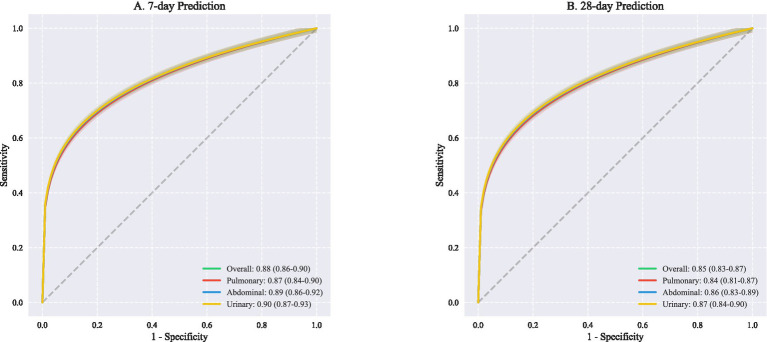
ROC curves for outcome prediction at different. This figure presents receiver operating characteristic (ROC) curves comparing predictive performance across different infection sources at two time points. **(A)** Shows 7-day prediction outcomes with overall AUROC of 0.88 (95% CI: 0.86–0.90), demonstrating excellent discriminative ability. Source-specific analyses revealed comparable performance: pulmonary infections (AUROC 0.87, 95% CI: 0.84–0.90), abdominal infections (AUROC 0.89, 95% CI: 0.86–0.92), and urinary infections (AUROC 0.90, 95% CI: 0.87–0.93). **(B)** Illustrates 28-day prediction performance, with slightly lower but still robust overall AUROC of 0.85 (95% CI: 0.83–0.87). Similar patterns were observed across infection sources: pulmonary (AUROC 0.84, 95% CI: 0.81–0.87), abdominal (AUROC 0.86, 95% CI: 0.83–0.89), and urinary tract infections (AUROC 0.87, 95% CI: 0.84–0.90). AUROC, Area under the receiver operating characteristic curve; CI, Confidence interval; ROC, Receiver operating characteristic.

Prediction accuracy showed temporal dependencies, with superior performance for short-term outcomes. The 7-day prediction model achieved higher discrimination (overall AUROC 0.88, 95% CI: 0.86–0.90) compared to the 28-day model (AUROC 0.85, 95% CI: 0.83–0.87). This advantage was particularly pronounced for urinary tract infections (7-day AUROC 0.90 vs. 28-day AUROC 0.87) (Figure 3), suggesting that early trajectory prediction may be most reliable for urinary source sepsis.

Additional analyses revealed robust model performance across different validation cohorts (Supplementary Figure 3) and patient subgroups (Supplementary Figure 4). Detailed statistical methods, including data preprocessing procedures and validation strategies, are provided in Supplementary Appendix 1. Model architecture specifications and implementation details are described in Supplementary Appendix 2. Complete validation results and sensitivity analyses are available in Supplementary Appendices 4, 5, respectively. The source code for all analyses has been deposited in a public repository and is described in Supplementary Appendix 3.

Extended baseline characteristics for the development and validation cohorts (Supplementary Table 1), treatment characteristics and clinical outcomes by recovery trajectory group (Supplementary Table 2) are provided in the Supplementary materials. These Supplementary materials offer comprehensive documentation of our analytical approach and additional evidence supporting the robustness of our findings.

## Discussion

This multi-cohort study identified three distinct sepsis recovery trajectories with unique immunological signatures and clinical outcomes. Our findings have several important implications for sepsis research and clinical practice. First, the identification of reproducible trajectory patterns across different healthcare settings suggests that sepsis recovery follows predictable pathways that may be amenable to early intervention. Second, the strong association between trajectory patterns and immunological profiles provides new insights into the biological mechanisms underlying different recovery patterns. Third, our machine learning model demonstrates that early trajectory prediction is feasible and could potentially guide personalized therapeutic strategies.

The immunological signatures we identified align with current understanding of sepsis pathophysiology while providing new insights into recovery patterns. The rapid recovery group’s balanced immune response, characterized by moderate inflammatory activation followed by timely resolution, represents an optimal host response to infection (14, 15). In contrast, the persistent inflammation observed in the slow recovery group and the profound immunosuppression in the deterioration group suggest distinct pathophysiological mechanisms that may require different therapeutic approaches (16, 17). These findings extend previous work by Seymour et al. (3) and van der Poll et al. (7) by demonstrating how immune dysfunction patterns evolve over time and correlate with clinical trajectories.

Temporal analysis revealed significant improvements in patient outcomes over the study period. The overall 28-day mortality decreased from 16.8% in 2014–2018 to 12.4% in 2019–2024, coinciding with updates to the Surviving Sepsis Campaign guidelines and more aggressive fluid resuscitation protocols. This improvement was consistent across all trajectory groups, though most pronounced in the rapid recovery group. Source-specific analysis demonstrated significant variations in outcomes (*p* < 0.001). In the deterioration group, mortality rates were highest for pulmonary infections (15.6%), followed by abdominal infections (13.8%), and lowest for urinary tract infections (12.4%). This pattern was consistent across all trajectory groups, suggesting that infection source may be an important determinant of recovery trajectory.

The robust performance of our prediction model across different healthcare settings represents a significant advance over existing prognostic tools (18, 19). Current severity scores such as SOFA and APACHE II provide static assessments but fail to capture the dynamic nature of sepsis recovery (5). Our model’s ability to predict trajectory membership within 12 h of admission, with consistent performance across external validation cohorts, suggests its potential utility for clinical decision-making (20). The identification of key predictive features, particularly early changes in SOFA scores and inflammatory markers, provides actionable insights for monitoring and risk stratification (21). Our findings suggest several modifiable factors that could improve outcomes based on predicted trajectories. The persistently elevated neutrophil-to-lymphocyte ratio in the deterioration group (19.0 [14.2–25.6]) suggests that modulation of the inflammatory-immune balance might be a therapeutic target. The low IL-6/IL-10 ratio observed in the rapid recovery group (1.8 [1.2–2.6]) could serve as a reference target for immunomodulatory interventions. For patients predicted to follow deterioration trajectories, early interventions might include more aggressive source control, enhanced hemodynamic monitoring, earlier initiation of immunomodulatory therapy, and intensified infection surveillance. The timing of interventions appears critical, with the greatest impact observed within the first 72 h. Standard of care evolution over the study period (2014–2024) showed improved outcomes across all trajectories, particularly with the implementation of rapid response protocols and standardized sepsis bundles. These improvements were most notable in centers that maintained strict adherence to updated treatment guidelines and implemented comprehensive monitoring protocols.

Several limitations should be considered when interpreting our findings. First, despite the large sample size and multi-center validation, our cohorts were primarily from high-income countries, potentially limiting generalizability to resource-limited settings (2). Second, while our immunological profiling was comprehensive, we could not measure all potentially relevant immune markers due to practical constraints (22). Third, the retrospective nature of our study precluded analysis of certain potentially important variables, such as genetic factors and pre-hospital care patterns (23).

These limitations notwithstanding, our findings have important implications for future research and clinical practice. The identification of distinct recovery trajectories suggests the need for trajectory-specific therapeutic approaches (24). For example, patients predicted to follow the deterioration trajectory might benefit from more aggressive initial interventions or novel immunomodulatory therapies (3, 4). Future randomized trials could use trajectory prediction to stratify patients and test trajectory-specific interventions.

## Conclusion

In conclusion, this study demonstrates that sepsis recovery follows distinct trajectories characterized by unique immunological signatures. Early identification of these trajectories through machine learning may enable more personalized therapeutic approaches and improve patient outcomes. Future studies should focus on validating these findings in prospective cohorts and developing trajectory-specific therapeutic strategies.

## Data Availability

The raw data supporting the conclusions of this article will be made available by the authors, without undue reservation.
