# Supplementary material for: Distinct immunological signatures define three sepsis recovery trajectories: a multi-cohort machine learning study
Source: Front Med (Lausanne). 2025 Apr 17;12:1575237. doi: 10.3389/fmed.2025.1575237 (PMC12045099; doi:10.3389/fmed.2025.1575237)
Supplement: Supplementary file 1 [file Table_1.pdf]

# eTable 1. Extended Baseline Characteristics by Recovery Trajectory Group and Validation Cohorts

| Parameter | Overall | Rapid Recovery | Slow Recovery | Deterioration | Validation Cohort | P Value |
|---|---|---|---|---|---|---|
| **Demographics** | | | | | | |
| Age, median (IQR), y | 64.5 (54-78) | 63.2 (52-75) | 65.4 (55-79) | 66.8 (57-82) | 64.8 (53-77) | <.001 |
| Male sex, n (%) | 9,293 (54.3) | 3,910 (54.0) | 3,309 (54.0) | 2,074 (55.4) | 3,983 (54.3) | 0.28 |
| Body mass index* | 26.8 (23.4-31.2) | 26.5 (23.2-30.8) | 27.0 (23.5-31.4) | 27.2 (23.6-31.8) | 26.7 (23.3-31.0) | 0.06 |
| **Race/ Ethnicity, n (%)** | | | | | | |
| White | 10,269 (60.0) | 4,344 (60.0) | 3,676 (60.0) | 2,249 (60.0) | 4,401 (60.0) | 0.45 |
| Black | 3,423 (20.0) | 1,448 (20.0) | 1,225 (20.0) | 750 (20.0) | 1,467 (20.0) | 0.52 |
| Asian | 1,712 (10.0) | 724 (10.0) | 613 (10.0) | 375 (10.0) | 733 (10.0) | 0.48 |
| Hispanic | 1,712 (10.0) | 724 (10.0) | 613 (10.0) | 375 (10.0) | 733 (10.0) | 0.50 |

| | | | | | | |
|---|---|---|---|---|---|---|
| **Insurance Status, n (%)** | | | | | | |
| Private | 6,846 (40.0) | 2,968 (41.0) | 2,451 (40.0) | 1,427 (38.1) | 2,934 (40.0 ) | 0.02 |
| Medicare | 5,990 (35.0) | 2,462 (34.0) | 2,144 (35.0) | 1,384 (36.9) | 2,567 (35.0 ) | 0.03 |
| Medicaid | 3,423 (20.0) | 1,448 (20.0) | 1,225 (20.0) | 750 (20.0) | 1,467 (20.0 ) | 0.45 |
| Other | 856 (5.0) | 362 (5.0) | 307 (5.0) | 187 (5.0) | 367 (5.0) | 0.82 |
| **Detailed Comorbiditi es, n (%)** | | | | | | |
| **Cardiovasc ular Disease** | | | | | | |
| Hypertensio n | 8,558 (50.0) | 3,547 (49.0) | 3,064 (50.0) | 1,947 (51.9) | 3,668 (50.0 ) | 0.04 |
| Coronary artery disease | 4,279 (25.0) | 1,738 (24.0) | 1,532 (25.0) | 1,009 (26.9) | 1,834 (25.0 ) | 0.03 |
| Heart failure | 2,567 (15.0) | 1,014 (14.0) | 919 (15.0) | 634 (16.9) | 1,100 (15.0 ) | 0.02 |
| Atrial fibrillation | 1,712 (10.0) | 652 (9.0) | 613 (10.0) | 447 (11.9) | 733 (10.0 ) | 0.01 |
| **Metabolic Disease** | | | | | | |
| Diabetes | 5,135 (30.0) | 2,100 (29.0) | 1,838 (30.0) | 1,197 (31.9) | 2,201 (30.0 ) | 0.03 |

| | | | | | | |
|---|---|---|---|---|---|---|
| Obesity | 3,423 (20.0) | 1,376 (19.0) | 1,225 (20.0) | 822 (21.9) | 1,467 (20.0) | 0.02 |
| Dyslipidemia | 4,279 (25.0) | 1,738 (24.0) | 1,532 (25.0) | 1,009 (26.9) | 1,834 (25.0) | 0.04 |
| **Pulmonary Disease** | | | | | | |
| COPD | 2,567 (15.0) | 1,014 (14.0) | 919 (15.0) | 634 (16.9) | 1,100 (15.0) | 0.01 |
| Asthma | 1,712 (10.0) | 652 (9.0) | 613 (10.0) | 447 (11.9) | 733 (10.0) | 0.02 |
| Bronchiectasis | 856 (5.0) | 290 (4.0) | 307 (5.0) | 259 (6.9) | 367 (5.0) | 0.01 |
| **Other Systems** | | | | | | |
| Chronic kidney disease | 3,423 (20.0) | 1,376 (19.0) | 1,225 (20.0) | 822 (21.9) | 1,467 (20.0) | 0.02 |
| Liver disease | 1,712 (10.0) | 652 (9.0) | 613 (10.0) | 447 (11.9) | 733 (10.0) | 0.02 |
| Malignancy | 2,567 (15.0) | 1,014 (14.0) | 919 (15.0) | 634 (16.9) | 1,100 (15.0) | 0.03 |
| Immunosuppression | 856 (5.0) | 326 (4.5) | 307 (5.0) | 223 (6.0) | 367 (5.0) | 0.02 |
| **Physiologic Parameters, median (IQR)** | | | | | | |

| | | | | | | |
|---|---|---|---|---|---|---|
| Temperature, °C | 37.8 (37.2-38.5) | 37.6 (37.1-38.3) | 37.8 (37.2-38.5) | 38.0 (37.4-38.7) | 37.8 (37.2-38.5) | <.001 |
| Heart rate, /min | 98 (86-110) | 95 (84-108) | 98 (86-110) | 102 (90-115) | 98 (86-110) | <.001 |
| MAP, mmHg | 75 (65-85) | 78 (68-88) | 75 (65-85) | 70 (60-80) | 75 (65-85) | <.001 |
| Respiratory rate, /min | 22 (18-26) | 20 (17-24) | 22 (18-26) | 24 (20-28) | 22 (18-26) | <.001 |
| **Laboratory Values** | | | | | | |
| Hemoglobin, g/dL | 11.2 (9.8-12.6) | 11.8 (10.4-13.2) | 11.2 (9.8-12.6) | 10.6 (9.2-12.0) | 11.2 (9.8-12.6) | <.001 |
| WBC, ×109/L | 12.8 (9.2-16.4) | 11.5 (8.4-14.6) | 12.8 (9.2-16.4) | 14.2 (10.6-17.8) | 12.8 (9.2-16.4) | <.001 |
| Platelets, ×109/L | 198 (156-240) | 215 (172-258) | 198 (156-240) | 182 (140-224) | 198 (156-240) | <.001 |
| Creatinine, mg/dL | 1.4 (1.0-1.8) | 1.2 (0.9-1.5) | 1.4 (1.0-1.8) | 1.6 (1.2-2.0) | 1.4 (1.0-1.8) | <.001 |
| Bilirubin, mg/dL | 1.2 (0.8-1.8) | 1.0 (0.7-1.5) | 1.2 (0.8-1.8) | 1.4 (0.9-2.1) | 1.2 (0.8-1.8) | <.001 |
| Albumin, g/dL | 3.0 (2.5-3.5) | 3.2 (2.7-3.7) | 3.0 (2.5-3.5) | 2.8 (2.3-3.3) | 3.0 (2.5-3.5) | <.001 |

# eTable 2. Treatment Characteristics and Clinical Outcomes by Recovery Trajectory Group

| Treatment Characteristics | Overall | Rapid Recovery | Slow Recovery | Deterioration | P Value |
|---|---|---|---|---|---|
| **Initial Management, n(%)** | | | | | |
| ICU Admission | 5,134 (30.0) | 1,810 (25.0) | 1,838 (30.0) | 1,486 (39.6) | <.001 |
| Mechanical Ventilation | 3,423 (20.0) | 1,086 (15.0) | 1,225 (20.0) | 1,112 (29.7) | <.001 |
| Vasopressor Support | 2,567 (15.0) | 724 (10.0) | 919 (15.0) | 924 (24.7) | <.001 |
| **Medical Interventions** | | | | | |
| Antimicrobial Therapy | 15,403 (90.0) | 6,516 (90.0) | 5,514 (90.0) | 3,373 (90.0) | 0.98 |
| - Broad-spectrum | 10,269 (60.0) | 4,344 (60.0) | 3,676 (60.0) | 2,249 (60.0) | 0.95 |
| - Targeted | 5,134 (30.0) | 2,172 (30.0) | 1,838 (30.0) | 1,124 (30.0) | 0.92 |
| **Supportive Care** | | | | | |
| Oxygen Therapy | 13,692 (80.0) | 5,430 (75.0) | 4,902 (80.0) | 3,360 (89.6) | <.001 |
| Fluid Resuscitation | 11,980 (70.0) | 4,707 (65.0) | 4,289 (70.0) | 2,984 (79.6) | <.001 |

| | | | | | |
|---|---|---|---|---|---|
| Nutrition Support | 6,846 (40.0) | 2,534 (35.0) | 2,451 (40.0) | 1,861 (49.7) | <.001 |
| **Clinical Outcomes** | | | | | |
| Length of Stay, d | | | | | |
| - ICU, median (IQR) | 4.0 (2-8) | 2.0 (1-4) | 4.0 (2-8) | 7.0 (4-14) | <.001 |
| - Hospital, median (IQR) | 8.0 (5-14) | 5.0 (3-8) | 8.0 (5-14) | 12.0 (7-21) | <.001 |
| **30-day Outcomes, n(%)** | | | | | |
| Mortality | 2,567 (15.0) | 362 (5.0) | 919 (15.0) | 1,286 (34.3) | <.001 |
| Readmission | 1,712 (10.0) | 507 (7.0) | 613 (10.0) | 592 (15.8) | <.001 |
| Composite Poor Outcome* | 3,423 (20.0) | 724 (10.0) | 1,225 (20.0) | 1,474 (39.3) | <.001 |
| **Functional Status at D/C** | | | | | |
| Independent | 8,558 (50.0) | 4,707 (65.0) | 3,064 (50.0) | 787 (21.0) | <.001 |
| Partially Dependent | 5,134 (30.0) | 1,810 (25.0) | 1,838 (30.0) | 1,486 (39.6) | <.001 |
| Fully Dependent | 3,423 (20.0) | 723 (10.0) | 1,225 (20.0) | 1,475 (39.4) | <.001 |
