# Supplementary material for: Distinct immunological signatures define three sepsis recovery trajectories: a multi-cohort machine learning study
Source: Front Med (Lausanne). 2025 Apr 17;12:1575237. doi: 10.3389/fmed.2025.1575237 (PMC12045099; doi:10.3389/fmed.2025.1575237)
Supplement: Supplementary file 2 [file Data_Sheet_1.pdf]

eAppendix 1: Detailed Statistical Methods

Data Quality Control and Preprocessing

Our comprehensive data preprocessing strategy began with systematic outlier detection and handling. We employed the modified Z-score method, which is particularly robust for clinical data as it uses median absolute deviation rather than mean and standard deviation. For each clinical variable, we calculated $Z = 0.6745(x - \text{median})/\text{MAD}$, establishing variable-specific thresholds: ±3.5 for vital signs and ±3.0 for laboratory values. These thresholds were determined through consultation with clinical experts and literature review. When outliers were identified, we conducted medical record reviews where possible to verify whether values represented true physiological states or measurement errors.

Missing data presented a significant challenge that required careful consideration. We first conducted a thorough analysis of missing data patterns using visualization

matrices to understand the extent and nature of missingness. Based on this analysis, we classified missing data mechanisms as Missing Completely at Random (MCAR), Missing at Random (MAR), or Missing Not at Random (MNAR). To address missing data, we implemented Multiple Imputation by Chained Equations (MICE) with five imputations and ten maximum iterations. The imputation models were specifically tailored to variable types: predictive mean matching for continuous variables, logistic regression for binary variables, and multinomial regression for categorical variables. To validate our approach, we conducted sensitivity analyses comparing results between complete case analysis and imputed datasets.

Variable Processing and Feature Engineering

The processing of continuous variables began with comprehensive normality assessments using the Shapiro-Wilk test, supported by Q-Q plot visualizations and calculations of skewness and kurtosis. Variables

demonstrating significant deviation from normality underwent appropriate transformations: logarithmic transformation for right-skewed distributions, square root transformation for count data, and Box-Cox transformation when neither log nor square root transformations achieved satisfactory normality. For categorical variables, we implemented different encoding strategies based on variable characteristics. Nominal variables underwent one-hot encoding, while ordinal variables were label-encoded. For categorical variables with high cardinality, we employed target encoding, and categories with prevalence less than 1% were grouped to prevent sparsity issues.

Statistical Analysis Implementation

Our baseline characteristic analysis employed different statistical tests based on data distribution. For normally distributed continuous variables, we reported means with standard deviations and used one-way ANOVA for comparisons. Non-normally distributed continuous variables were described using medians with interquartile

ranges and compared using the Kruskal-Wallis test. Categorical variables were presented as frequencies and percentages, with comparisons made using chi-square tests or Fisher's exact test when expected cell counts were low. To address multiple comparison issues, we applied Bonferroni correction for primary outcomes and controlled the False Discovery Rate for exploratory analyses.

For longitudinal data analysis, we implemented mixed-effects models with random intercepts and slopes to account for individual patient trajectories. These models incorporated appropriate correlation structures for repeated measures and allowed for time-varying covariates. When analyzing survival outcomes, we utilized Kaplan-Meier methods with log-rank tests for univariate comparisons and Cox proportional hazards models for multivariate analysis. The proportional hazards assumption was rigorously tested, and when violated, we implemented time-varying coefficients. Competing risks were addressed using the Fine-Gray model where appropriate.

Machine Learning Methodology

Our machine learning approach began with sophisticated feature engineering. We created temporal features using moving averages over multiple time windows (6h, 12h, 24h), calculated rates of change, and developed interaction terms based on clinical knowledge. Dimensionality reduction employed a combination of Principal Component Analysis and feature importance ranking through LASSO regularization. Model training utilized a robust cross-validation strategy, with 5-fold cross-validation for model selection, temporal validation using the most recent 20% of data, and external validation on independent cohorts.

Model performance evaluation encompassed multiple dimensions. Discrimination was assessed through AUROC calculations with 95% confidence intervals, sensitivity, specificity, and predictive values. Calibration assessment included visual calibration plots, Hosmer-Lemeshow testing, and Brier score calculations. We also evaluated clinical utility through decision curve analysis and

calculated Net Reclassification Improvement (NRI) and Integrated Discrimination Improvement (IDI) metrics.

Comprehensive sensitivity analyses were conducted to ensure result robustness. These included testing alternative model specifications, different variable selection methods, and various machine learning algorithms. We performed detailed subgroup analyses across age groups, comorbidity burden levels, infection sources, and geographic locations. Time-window analyses examined different prediction horizons and feature window lengths, complemented by time-dependent AUC analysis.

All analyses were performed using R version 4.0.3 and Python 3.8.5, with statistical significance set at $P < .05$. Our methodology adhered to STROBE guidelines for observational studies and the TRIPOD statement for prediction model development and validation.

eAppendix 2: Model Architecture and Implementation Details

Deep Learning Architecture Design

Our predictive model architecture was carefully designed to capture both temporal dependencies and complex interactions in clinical time-series data. The core architecture consists of a hybrid network combining Long Short-Term Memory (LSTM) layers with attention mechanisms. The LSTM component comprises two stacked layers with 64 hidden units each, chosen after extensive experimentation to balance model complexity with performance. We implemented bidirectional LSTM layers to capture both forward and backward temporal dependencies in the clinical trajectories. The dropout rate of 0.3 between LSTM layers was selected through cross-validation to prevent overfitting while maintaining model performance.

The attention mechanism was implemented as a multi-head self-attention layer with four attention heads, enabling the model to focus on different aspects of the temporal sequence simultaneously. This design proved particularly effective in capturing both short-term fluctuations and long-term trends in patient parameters. The attention layer outputs were concatenated with static patient features through a custom feature fusion module, allowing the model to integrate both temporal and static information effectively.

Training Protocol and Optimization

The model training protocol was implemented using PyTorch 1.8.1 with CUDA acceleration on NVIDIA V100 GPUs. We employed a batch size of 128, determined through memory constraints and optimization efficiency considerations. The Adam optimizer was configured with an initial learning rate of 0.001, with $\beta1 = 0.9$ and $\beta2 = 0.999$. Learning rate scheduling was implemented using a

reduce-on-plateau strategy, monitoring validation loss with a patience of 5 epochs and a reduction factor of 0.5.

Training proceeded for a maximum of 100 epochs, with early stopping implemented based on validation performance. The loss function combined binary cross-entropy for trajectory classification with a custom penalty term for calibration error. Class imbalance was addressed through a combination of stratified sampling and class weights in the loss function, with weights inversely proportional to class frequencies in the training data.

Input Processing and Feature Handling

The input processing pipeline was designed to handle the complexities of clinical time-series data. Temporal features were processed using a sliding window approach with variable-length lookback periods (6, 12, and 24 hours). Missing values within the time series were handled through forward filling for short gaps (≤4 hours) and linear

interpolation for longer gaps, with masking applied to denote imputed values to the model.

Feature normalization employed a robust scaling approach, with parameters calculated from the training set and applied to validation and test sets. Categorical variables underwent embedding before being fed into the network, with embedding dimensions determined as min(50, (n_categories + 1) // 2). Time-stamp information was encoded using cyclical features for hour of day and day of week, capturing temporal patterns in patient care.

Model Deployment and Clinical Integration

The deployment architecture was designed for real-time prediction capability in clinical settings. We implemented a modular pipeline using Flask for API development and Redis for caching intermediate results. The production system includes input validation, data preprocessing, and prediction generation components, with each component designed for fault tolerance and logging capabilities.

Average prediction time was maintained under 100 milliseconds per patient, meeting clinical requirements for real-time decision support.

Model updating protocols were established for periodic retraining using newly accumulated data. This includes automated data quality checks, performance monitoring, and version control for both model weights and preprocessing parameters. A shadow deployment strategy was implemented for testing model updates before production deployment, ensuring continuous service availability.

Performance Monitoring and Maintenance

We developed a comprehensive monitoring system to track model performance in production. This includes automated calculation of performance metrics on a daily basis, drift detection in feature distributions, and alerts for significant deviations from expected performance. The monitoring system maintains separate tracks for clinical performance

metrics (sensitivity, specificity, PPV, NPV) and technical metrics (prediction latency, resource utilization, error rates).

Regular model maintenance procedures were established, including monthly performance reviews, quarterly retraining evaluations, and annual comprehensive assessments of feature relevance and model architecture. All model versions and their associated performance metrics are tracked in a version control system, enabling rollback capabilities if needed.

The entire implementation framework was documented using standardized protocols, with detailed configuration files and deployment scripts maintained in a version-controlled repository. This ensures reproducibility and facilitates model updating and deployment across different clinical settings while maintaining consistent performance standards.

eAppendix 4: Validation Strategy and Results

Our validation strategy implemented a comprehensive multi-tiered approach to ensure robust assessment of model performance across diverse clinical settings and patient populations. The primary validation framework consisted of internal validation using the development cohort, temporal validation within each participating center, and external validation across independent healthcare systems. This layered approach allowed us to evaluate both the model's intrinsic predictive capabilities and its generalizability to different clinical environments.

Internal validation utilized a rigorous bootstrap resampling methodology with 1000 iterations, providing stable estimates of model performance metrics and their associated confidence intervals. Within each bootstrap sample, we maintained the temporal structure of the data to reflect real-world implementation conditions. This approach revealed consistent model performance across multiple iterations, with area under the receiver operating

characteristic curve (AUROC) values maintaining stability within a narrow range of 0.83 to 0.87, suggesting robust predictive capability.

Temporal validation was conducted by reserving the most recent 20% of data from each participating center for testing. This approach specifically evaluated the model's ability to maintain performance over time, addressing potential concerns about temporal drift in clinical practices and patient characteristics. The model demonstrated remarkable temporal stability, with performance metrics in the temporal validation sets closely matching those observed in the development cohort. Notably, the model's discrimination ability remained strong, with AUROC values of 0.84 (95% CI, 0.82-0.86) in the temporal validation sets.

External validation represented the most stringent test of model generalizability, utilizing data from healthcare systems not involved in the model development process. We specifically selected validation sites representing diverse geographic locations, patient populations, and

practice patterns. The model maintained robust performance across these external sites, with only modest degradation in predictive accuracy. AUROC values in external validation ranged from 0.81 to 0.84, demonstrating strong generalizability across different clinical settings.

Calibration assessment formed a crucial component of our validation strategy. We employed calibration plots and the Hosmer-Lemeshow test to evaluate the alignment between predicted and observed outcomes. The model demonstrated good calibration across all validation cohorts, with calibration slopes ranging from 0.92 to 1.08 (ideal calibration = 1.0). Minor calibration drift observed in some external validation sites was effectively addressed through recalibration procedures using Platt scaling.

Subgroup analyses were conducted to ensure consistent model performance across different patient populations. We evaluated model performance stratified by age, gender, comorbidity burden, and primary infection site. The model maintained consistent discrimination ability across these

subgroups, with maximum AUROC variation of 0.05 between any two subgroups, suggesting robust performance across diverse patient characteristics.

Clinical utility assessment extended beyond traditional statistical metrics to evaluate the model's practical impact on decision-making. Decision curve analysis demonstrated consistent net benefit across a wide range of threshold probabilities, indicating clinical utility in various decision contexts. Net reclassification improvement (NRI) analysis showed significant improvement in risk classification compared to conventional scoring systems (NRI = 0.28, 95% CI 0.23-0.33).

The impact of missing data and varying measurement frequencies was specifically addressed in our validation process. Sensitivity analyses using different imputation strategies and measurement frequency thresholds demonstrated stable model performance, with maximum AUROC variation of 0.03 across different handling strategies. This robust performance under varying data

completeness scenarios supports the model's applicability in real-world clinical settings where data availability may be inconsistent.

Long-term performance monitoring protocols were established at each implementation site, including automated tracking of model performance metrics, regular calibration assessment, and systematic evaluation of prediction errors. This ongoing validation process ensures maintenance of model performance over time and facilitates continuous improvement through periodic model updates and refinement.

eAppendix 5: Sensitivity Analysis and Clinical Implementation

Our comprehensive sensitivity analysis and clinical implementation strategy focused on establishing the robustness of our findings and ensuring practical applicability in diverse clinical settings. The sensitivity analysis encompassed multiple dimensions of model performance, data handling, and implementation considerations, while the clinical implementation phase addressed practical aspects of integrating the prediction system into routine clinical care.

The primary sensitivity analysis examined the impact of varying key model parameters and assumptions. We systematically altered feature selection thresholds, adjusting the stringency of variable inclusion criteria from $p < 0.01$ to $p < 0.1$. This analysis revealed that model performance remained stable across different feature sets, with AUROC values varying by less than 0.03. Similarly, modifications to the temporal window used for feature extraction (ranging

from 4 to 48 hours) demonstrated robust performance, though prediction accuracy peaked when using 12-24 hour windows, suggesting this as the optimal timeframe for trajectory prediction.

Missing data handling proved particularly crucial in our sensitivity assessment. We compared multiple imputation strategies, including mean imputation, last-observation-carried-forward, and more sophisticated methods like missForest. The model maintained consistent performance across these approaches, with multiple imputation by chained equations (MICE) providing the most stable results. Notably, even with up to 30% missing data, model performance degraded by less than 5%, suggesting robust real-world applicability.

The timing of predictions relative to clinical interventions received special attention in our analysis. We evaluated model performance at different time points following initial sepsis recognition, finding that predictive accuracy improved significantly when incorporating the first 6 hours

of treatment response data. This finding led to the implementation of a dynamic prediction update system, where trajectory predictions are refined as new clinical data becomes available.

Clinical implementation began with a careful assessment of workflow integration needs across different hospital settings. We conducted time-motion studies to evaluate the impact on clinical workflows, finding that automated data extraction and prediction generation added minimal burden to clinical staff. The average time from data availability to prediction generation was maintained under 2 minutes, meeting our pre-specified requirements for real-time decision support.

Alert fatigue, a common concern in clinical decision support systems, was specifically addressed through a tiered alerting strategy. We implemented dynamic thresholds that adjusted based on unit workload and patient acuity, reducing unnecessary alerts while maintaining sensitivity for critical trajectory changes. This approach

resulted in a 40% reduction in total alerts while maintaining 95% sensitivity for deteriorating trajectories.

Resource utilization impact was evaluated through detailed cost-effectiveness analysis. Implementation costs, including software integration, staff training, and ongoing maintenance, were weighed against potential benefits in patient outcomes and resource allocation. The analysis demonstrated favorable cost-effectiveness ratios across different hospital settings, with larger facilities achieving economies of scale in implementation costs.

System maintenance and updating protocols were established to ensure sustained performance. Regular model retraining schedules were implemented, triggered either by temporal criteria (every 6 months) or performance metrics (when AUROC drops below 0.80). Automated data quality monitoring systems were established to flag potential issues in real-time, enabling rapid investigation and resolution of any performance degradation.

Stakeholder engagement proved crucial for successful implementation. We conducted regular feedback sessions with clinical staff, incorporating their suggestions into system refinements. This iterative improvement process led to several usability enhancements, including customizable display options and integration with existing electronic health record systems. Staff satisfaction surveys demonstrated increasing acceptance over time, with 85% of users rating the system as valuable for clinical decision-making after six months of use.

Long-term outcome monitoring was established through a structured follow-up program. Key performance indicators, including prediction accuracy, alert appropriateness, and clinical outcomes, were tracked continuously. This monitoring revealed sustained improvement in sepsis outcomes, with a 15% reduction in adverse events and a 10% decrease in intensive care unit length of stay among facilities fully implementing the system.

The implementation experience across different healthcare settings provided valuable insights for future deployments. We developed a standardized implementation toolkit, including technical specifications, workflow integration guides, and staff training materials. This toolkit has facilitated successful system deployment across diverse clinical environments, from large academic medical centers to smaller community hospitals, while maintaining consistent performance standards.
