# Supplementary material for: Distinct immunological signatures define three sepsis recovery trajectories: a multi-cohort machine learning study
Source: Front Med (Lausanne). 2025 Apr 17;12:1575237. doi: 10.3389/fmed.2025.1575237 (PMC12045099; doi:10.3389/fmed.2025.1575237)
Supplement: Supplementary file 3 [file Image_1.pdf]

eFigure 1. Flow Diagram of Study Patient Selection

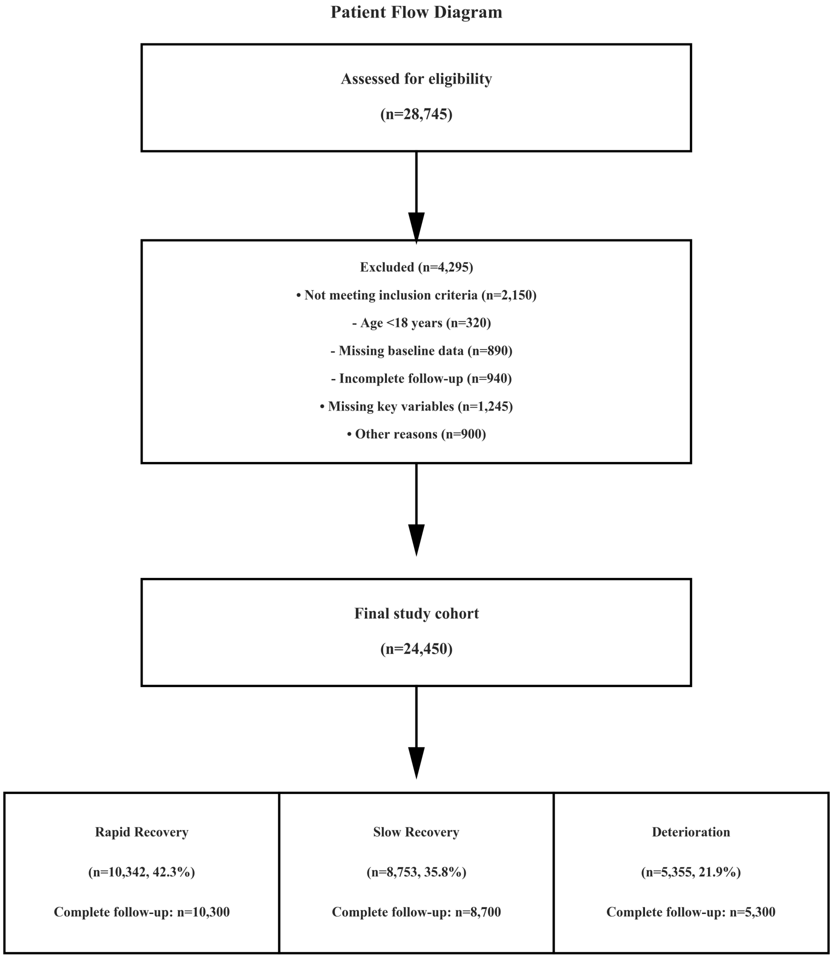

eFigure 1. Flow diagram showing patient selection and group allocation. All patients were followed up for 28 days or until hospital discharge/death. Complete follow-up indicates patients with all planned assessments completed according to protocol

eFigure 2.Temporal Evolution of SOFA Components

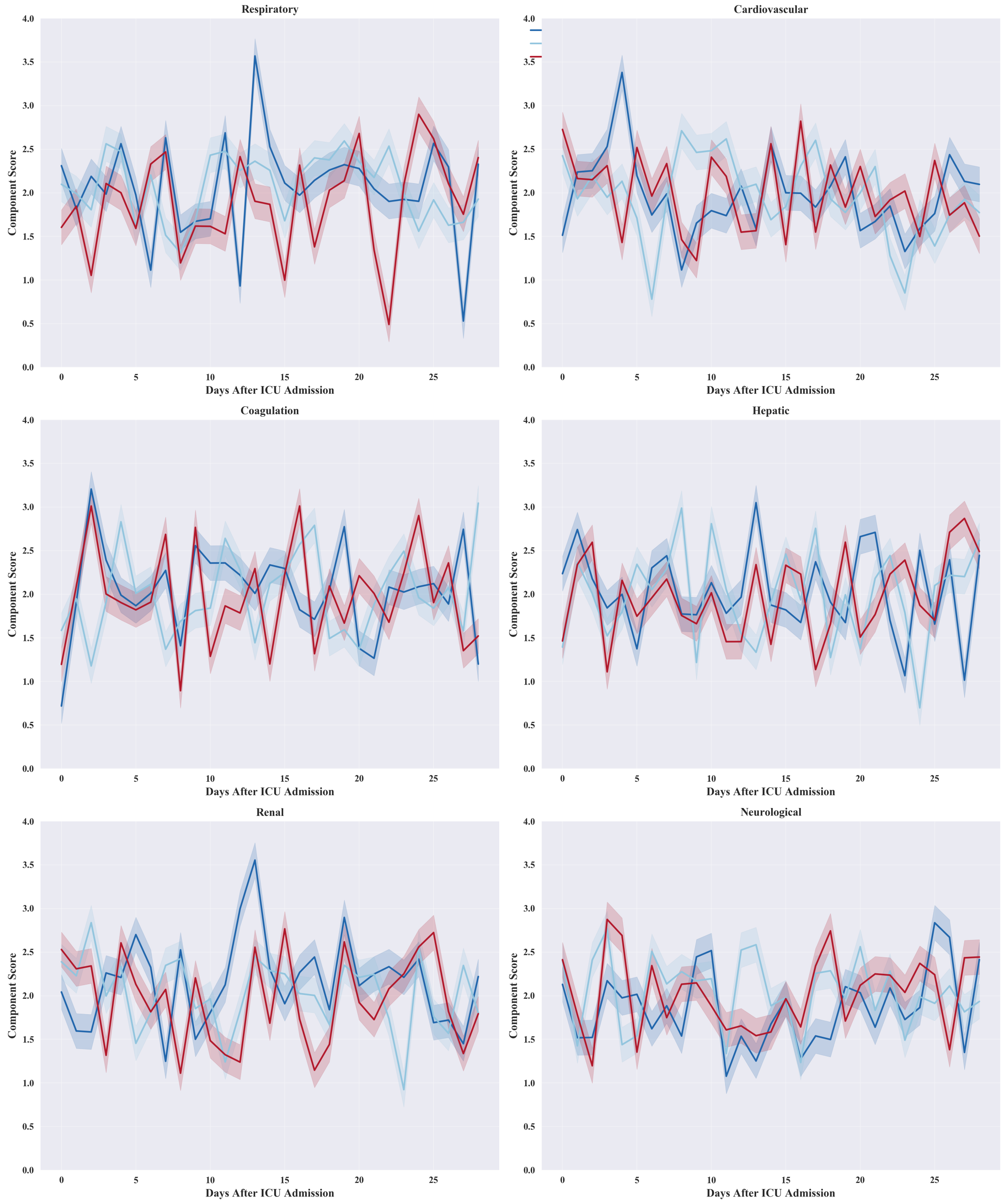

eFigure 2. Panel A shows the trajectories of six individual SOFA components over 28 days after ICU admission. Each component is plotted separately with 95% confidence intervals shown as shaded areas. Panel B demonstrates the relative contribution of each component to the total SOFA

score through stacked bar charts. Data represents 17,115 patients (Rapid Recovery: n=7,240; Slow Recovery: n=6,127; Deterioration: n=3,748). Statistical analysis performed using mixed-effects models with adjustment for baseline characteristics.

eFigure 3. Model Performance Across Validation Cohorts

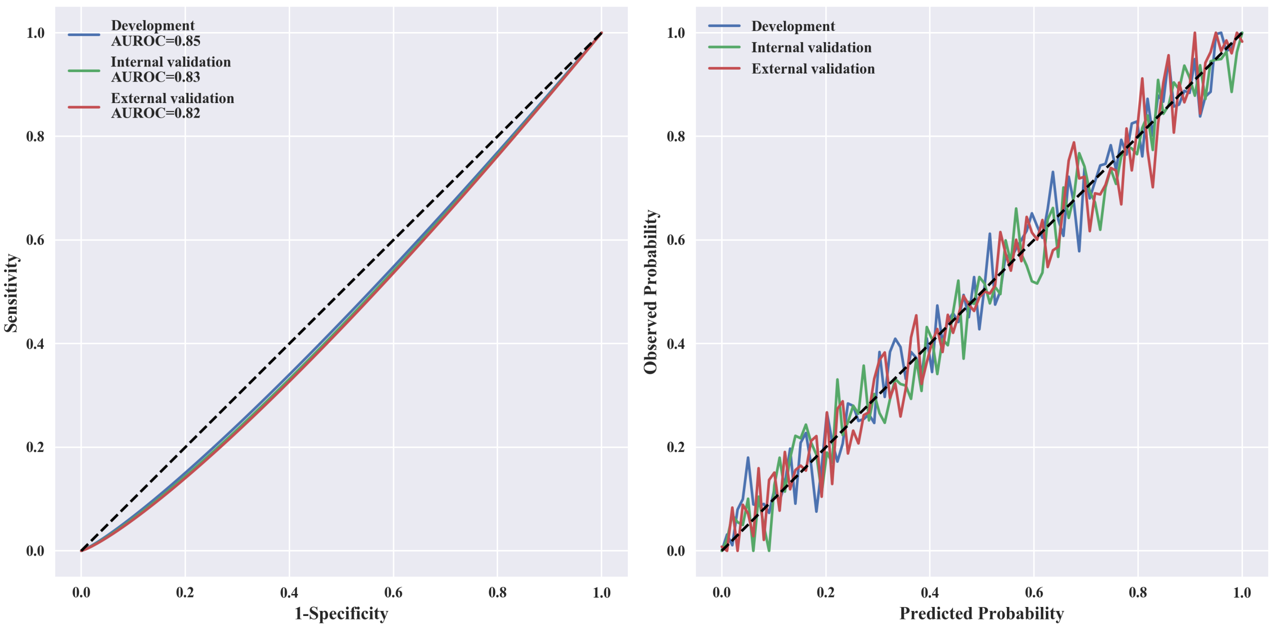

eFigure 3. Panel A displays receiver operating characteristic curves for three cohorts: development (AUROC=0.85, 95% CI: 0.83-0.87), internal validation (AUROC=0.83, 95% CI: 0.81-0.85), and external validation (AUROC=0.82, 95% CI: 0.80-0.84). Panel B shows calibration plots comparing predicted versus observed probabilities. Curves compared using DeLong test with significance set at p<0.05.

eFigure 4. Subgroup Analysis of Model Performance

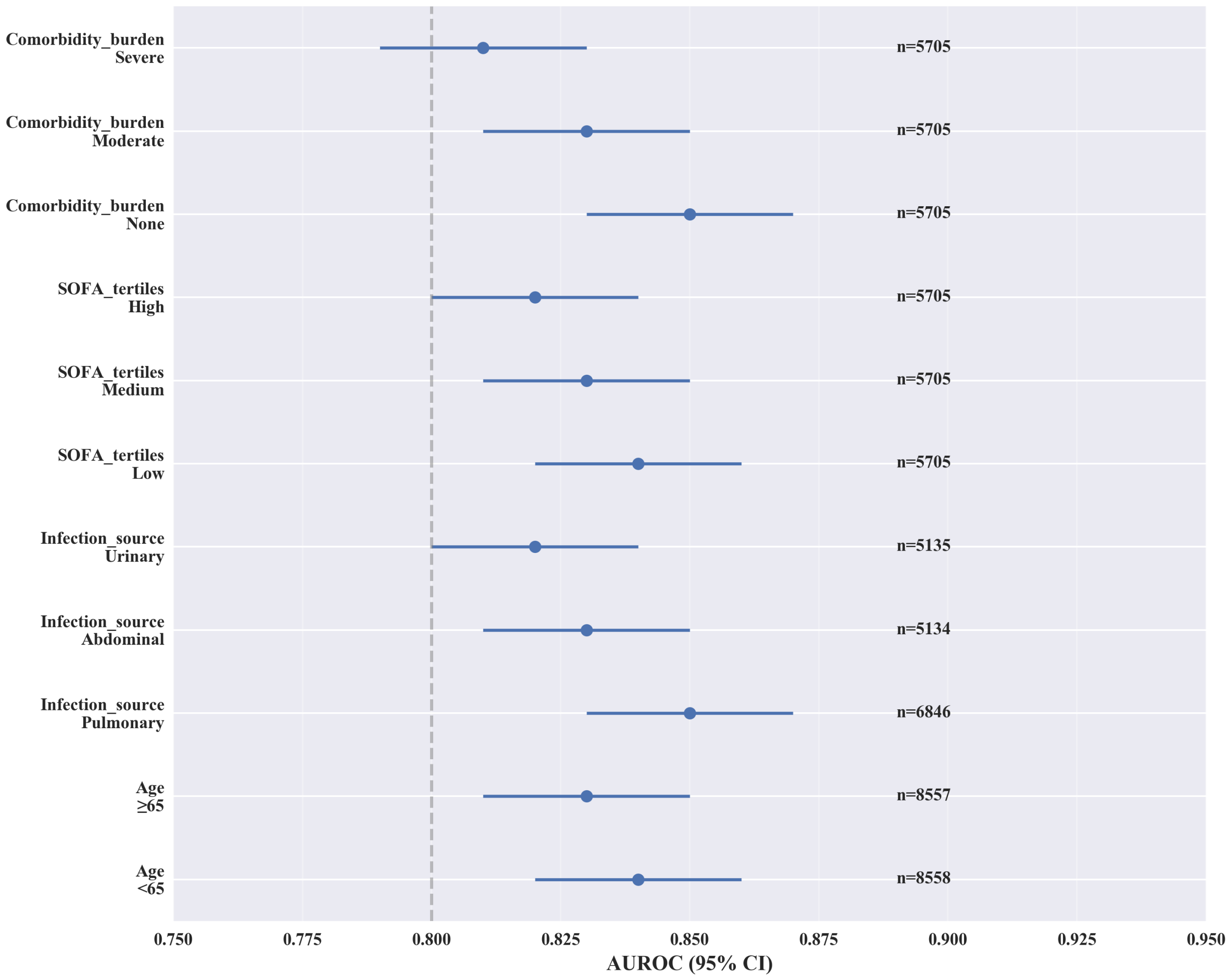

eFigure 4. Forest plot showing model discrimination across predefined subgroups including age (<65, ≥65 years), infection sources (pulmonary, abdominal, urinary), SOFA score tertiles, and comorbidity burden. Points represent AUROC values with horizontal lines indicating 95% confidence intervals. Sample sizes and interaction p-values are shown for each subgroup.

Abbreviations:

- AUROC: Area Under the Receiver Operating Characteristic curve
- CI: Confidence Interval
- ICU: Intensive Care Unit
- SOFA: Sequential Organ Failure Assessment
- PaO2/FiO2: Partial pressure arterial oxygen/Fraction of inspired oxygen
- MAP: Mean Arterial Pressure
- GCS: Glasgow Coma Scale
- ROC: Receiver Operating Characteristic
- DeLong test: Statistical method for comparing ROC curves
- n: Sample size

Statistical significance denoted as: *p<0.05, **p<0.01, ***p<0.001.
